# Quinia in Typhoid, and All Other Fevers

**Published:** 1845-08-01

**Authors:** 


### EDITORIAL, MEDICAL INTELLIGENCE, &c.

*Quinia in Typhoid, and all other Fevers.*—Prof. Mitchell, of Transylvania university, in a communication published in the Western Lancet, for May, 1845, advocates the free use of Quinine, not only in remittents, but in typhoid, typhus, congestive, yellow, "and, it may be, all the fevers named in the books." To quote his language —"the position I assume here, is plainly, and boldly, this: *there is but one feature, or element in either of the fevers named that is essential to its pathology, and that feature or property, or element bows before the potent sway of the* Sulphate of Quinine." He gives several instances illustrating the practical application of these views. It is to be regretted however, that they are not more numerous, and given at length, with more detail. We should, also, have been glad to have had a more full and explicit account of the doses which he is accustomed to administer, together with the circumstances affecting the quantity of the dose and its repetitions. He recommends its endermic application in cases in which it is liable to offend the stomach, by means of a cerate, containing it, which is applied to a blistered surface.

Prof. M. is certainly correct when he intimates that the views which he has thrown out in the article alluded to, will strike the minds of a considerable portion of the profession with surprise. We are, however, prepared for them, having been aware of the fact that they are entertained, and practically carried out by many western Practitioners. We have been informed that some who have adopted these views have found them so eminently valuable as means of successful practice, that they have endeavored to appropriate the great advantages which they are supposed to give, by maintaining secrecy, and professing to have discovered a therapeutical method peculiar to themselves. Indeed, we have been told that not only in fever distinguished as idiopathic, but even in the febrile movement symptomatic of local inflammation, this remedy is appropriate and potent in its influence; and that it is by some practitioners freely given under these circumstances with the best results. We have ourselves recently witnessed a single case of pneumonitis, in which in the active stage of the disease, it being determinable both by rational and physical signs, ten grains of Quinia were prescribed in the morning and repeated at evening, for two successive days, and in the mean time, or immediately following, the febrile symptoms became strikingly less, and the general condition of the patient greatly improved. We are by no means prepared to deny that the amendment in this case was merely a coincidence. A single fact of this kind, of course, affords no occasion for a positive deduction. Negatively, however, this single case is valuable. It goes to prove, at least, quite conclusively that the remedy may be given in large doses, under such circumstances, not only with impunity, but without interfering with the march of the disease toward a favorable termination. This is, certainly, a fact of not a little importance.

Having had some experience with the use of Quinia in large doses, in the treatment of Intermittents; having recommended its administration with boldness and freedom in Remittents under certain qualifications; and having expressed our belief, that acute local inflammation coincident with Intermittents constitutes no solid objection to its use in sufficient quantity to effect the specific object: (see Am. Jour. Med. Sciences, new series, vol. 11. p. 244) we are perhaps more accessible to views which would extend its useful application over a much wider sphere. But in making this remark we do not wish to be understood as concurring with the views entertained by Prof. M. and others. We are sufficiently impressed with their interest and importance to desire the collection and publication of facts, to serve as data for some positive deductions. We wish to have a series of careful observations, made by competent observers. If Prof. M. or others have not now the materials, they might be easily collected in a short period, with a little effort. Let a sufficient number of cases of the different varieties of fevers, distinguished as continued fevers, be submitted to the therapeutical principles of which we are speaking, and carefully recorded; we shall then have definite, palpable results, upon which to base positive conclusions. Until these data are furnished, it would be absurd and unphilosophical to form an opinion ; the evidence, however, which the article of Prof. M. presents, should suffice to authorize a trial of his principles. Let this be done by different individuals, and records carefully made, and the results will, if brought together, probably soon suffice, either to establish a truth which will prove of immense practical importance, or, on the other hand, to disprove a doctrine, which, if not true, can hardly fail to be pernicious.

